# Clinicopathological and molecular characteristics of primary pulmonary choriocarcinoma: a case report and systematic review

**DOI:** 10.3389/fonc.2026.1884668

**Published:** 2026-06-30

**Authors:** Yixin Liu, Xin Lv, Shu Su, Fangjun Chen, Lifeng Wang

**Affiliations:** Department of Oncology, Nanjing Drum Tower Hospital, Affiliated Hospital of Medical School, Nanjing University, Nanjing, China

**Keywords:** case report, immunotherapy, next-generation sequencing, primary pulmonary choriocarcinoma, systematic review

## Abstract

Primary pulmonary choriocarcinoma (PPC) is an exceptionally rare and aggressive non-gestational trophoblastic malignancy for which no standard treatment has been established. We report a 61-year-old man with PPC and brain metastases who received intracranial radiotherapy and first-line albumin-bound paclitaxel plus cisplatin, followed by etoposide, capecitabine, and camrelizumab after disease progression. Targeted next-generation sequencing identified loss-of-function or deleterious alterations involving *TP53*, *STK11*, *KEAP1*, and *SMARCA4*. The second-line immunotherapy-based combination was associated with prolonged extracranial disease control and radiological improvement, although the independent contribution of immune checkpoint inhibition could not be isolated from concurrent chemotherapy and radiotherapy; the disease later progressed intracranially, and the patient ultimately died of intra-abdominal hemorrhage with hepatic metastases. A systematic review of English-language PubMed reports from 2001 to 2026 identified 45 previously reported PPC patients, underscoring the rarity, diagnostic difficulty, aggressive course, and limited evidence base for this disease. Together, this case and literature synthesis suggest that PPC may be clinically and molecularly distinct from gestational choriocarcinoma; however, because these molecular observations derive from a single patient, they should be regarded as hypothesis-generating rather than establishing a general molecular signature of PPC, and that multimodal treatment incorporating immune checkpoint inhibition warrants further investigation in collaborative rare-tumor cohorts.

## Introduction

Choriocarcinoma is a malignant trophoblastic tumor characterized by cytotrophoblastic and syncytiotrophoblastic differentiation and secretion of human chorionic gonadotropin (hCG) (1). Most cases are gestational and arise from trophoblastic tissue after pregnancy. Although gonadal choriocarcinoma commonly metastasizes to the lung, primary pulmonary choriocarcinoma (PPC) is extremely uncommon, has nonspecific clinical and imaging features, and is often diagnosed only after pathological confirmation. Because PPC is highly invasive, prone to early metastasis, and lacks a recognized standard treatment, previous reviews have suggested that surgery combined with chemotherapy may be beneficial when complete resection is feasible (2). However, many patients present with unresectable or metastatic disease, and evidence for subsequent systemic therapy remains limited.

The biological distinction between PPC and gestational choriocarcinoma remains incompletely defined. Gestational choriocarcinoma is generally chemotherapy-sensitive and has a favorable prognosis when treated appropriately, whereas PPC often progresses rapidly and responds poorly to conventional chemotherapy. Molecular data are scarce, and only a small number of published PPC cases have included genomic profiling (3). We therefore report a male patient with advanced PPC who underwent targeted next-generation sequencing and received an immunotherapy-based second-line regimen. We also summarize the clinical characteristics, treatments, and outcomes of previously published English-language PPC cases to clarify the current evidence base and remaining therapeutic uncertainty.

## Case presentation

**Figure 1:**
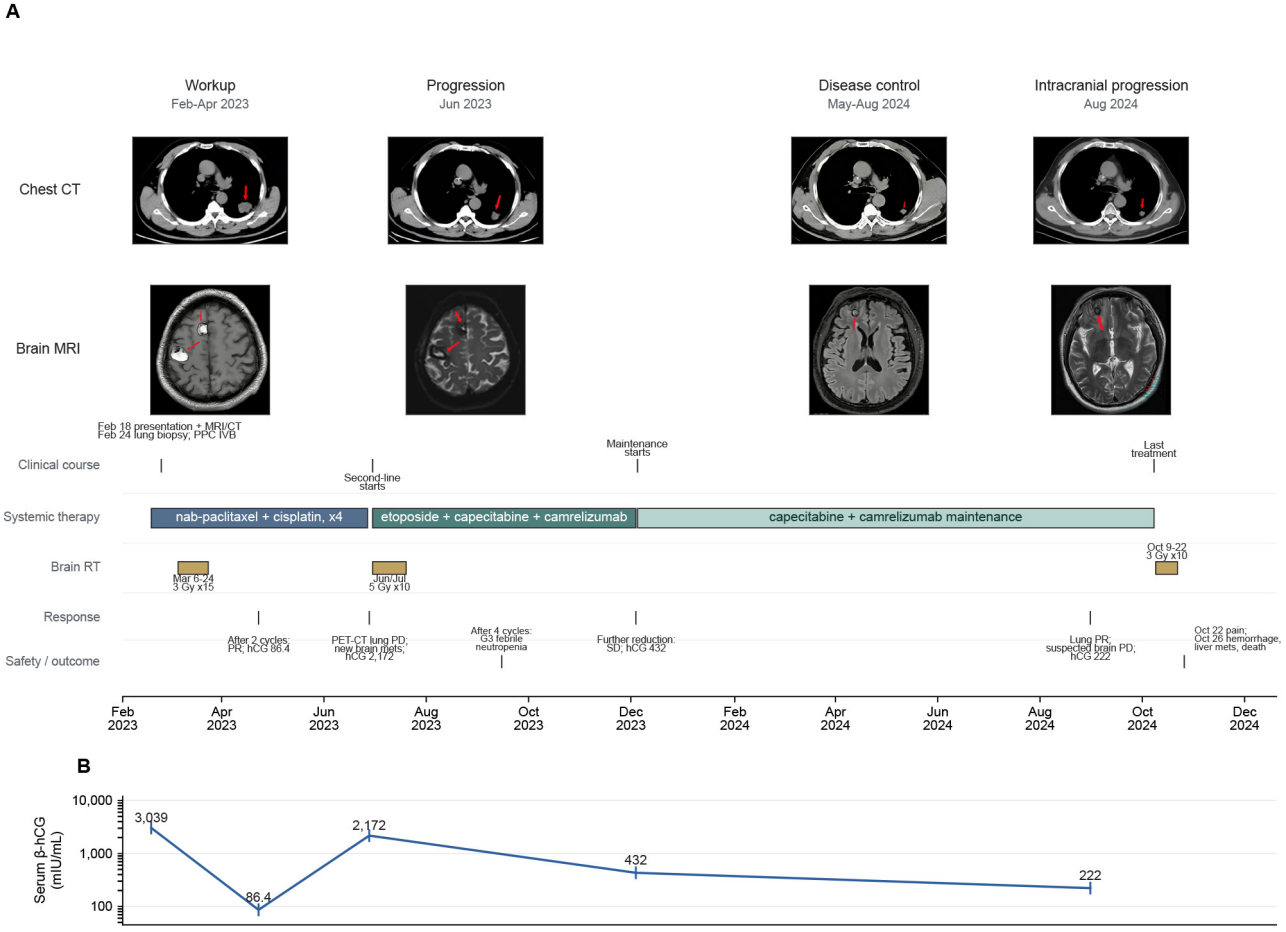
Treatment course, serum hCG dynamics, and radiological assessment. Serum hCG levels were monitored longitudinally during treatment. The treatment timeline shows first-line albumin-bound paclitaxel plus cisplatin followed by second-line etoposide, camrelizumab, and capecitabine. Representative chest CT and brain MRI images are shown below the timeline; red arrows indicate pulmonary lesions and intracranial metastases. **(A)** Treatment course and radiological assessment. **(B)** Serum hCG dynamics during treatment.

A 61-year-old man with a smoking history of more than 40 years presented on February 18, 2023, with left eyelid ptosis, headache, facial spasm, and dysarthria. Contrast-enhanced brain magnetic resonance imaging (MRI) showed multiple lesions in the right cerebral hemisphere, consistent with brain metastases, with the largest lesion measuring 28 x 24 mm. Contrast-enhanced computed tomography (CT) of the chest and abdomen identified a 41 x 31 mm mass in the lower lobe of the left lung with mild bilateral pleural thickening (**Figure 1**).

**Figure 2:**
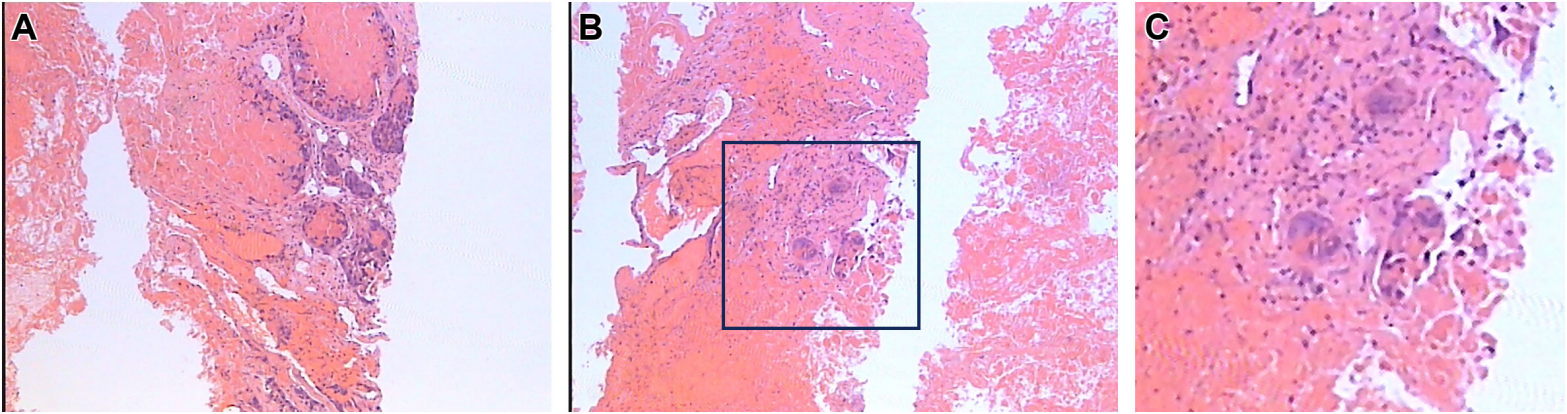
Histopathological examination of the lung biopsy (hematoxylin and eosin staining). **(A, B)** Low-power fields showing a poorly differentiated malignant tumor with extensive hemorrhage and necrosis. **(C)** Enlarged view of the boxed region in **(B)**, showing atypical mononuclear and multinucleated trophoblastic tumor cells admixed with hemorrhage, consistent with choriocarcinoma.

CT-guided biopsy of the lung lesion was performed on February 24, 2023. Pathological examination showed a poorly differentiated malignant tumor with extensive necrosis (**Figures 2A, B**). Higher-power examination demonstrated atypical mononuclear and multinucleated trophoblastic tumor cells admixed with extensive hemorrhage and necrosis (**Figure 2C**). Immunohistochemistry supported choriocarcinoma, with positivity for CK, c-Met, beta-hCG, and CK7; focal positivity for P40; and negativity for Syn, CgA, CD56, TTF-1, NUT, S100, CK20, Villin, and Napsin A. The Ki-67 index was approximately 90% in hot-spot areas. Serum beta-hCG was markedly elevated at 3, 039 IU/L, whereas scrotal and penile ultrasonography showed no evidence of a gonadal primary tumor. Contrast-enhanced computed tomography of the chest and abdomen demonstrated no mediastinal, retroperitoneal, or other extragonadal mass to suggest an alternative germ-cell primary, supporting a pulmonary origin. The patient was diagnosed with PPC of the left lung, staged as T2N0M1c, stage IVB.

After multidisciplinary review and exclusion of treatment contraindications, radiotherapy for intracranial metastatic lesions was initiated on March 6, 2023, targeting two right parietal lesions at a prescribed dose of 3 Gy × 15 fractions. Systemic therapy consisted of albumin-bound paclitaxel (200 mg on days 1 and 8) plus cisplatin (40 mg on days 1-3), repeated every 3 weeks. After two cycles, the response was assessed as partial response (PR), and serum beta-hCG decreased to 86.4 mIU/mL. After two additional cycles, PET-CT on June 28, 2023 showed progression of the pulmonary lesion, brain MRI revealed new metastatic lesions, and serum beta-hCG increased to 2, 172 mIU/mL. The disease was assessed as progressive disease (PD).

Second-line treatment was initiated on June 30, 2023, with etoposide (200 mg on days 1-3), camrelizumab (200 mg on day 1), and capecitabine (1.5 g twice daily on days 1-14), repeated every 3 weeks. Concurrent radiotherapy was administered for newly detected intracranial lesions at 5 Gy × 10 fractions. After two cycles, pulmonary and intracranial lesions decreased in size, and the response was assessed as stable disease. After two additional cycles, the patient developed grade 3 febrile neutropenia, which improved after supportive treatment.

On December 4, 2023, serum hCG had declined to 432 mIU/mL, and imaging showed further reduction of both pulmonary and intracranial lesions. Etoposide was discontinued on December 5, 2023, and maintenance capecitabine plus camrelizumab was continued. On August 31, 2024, contrast-enhanced chest CT showed further shrinkage of the pulmonary lesion, whereas brain imaging showed increased edema in the right frontal lesion and new enhancement near the right ventricular angle, suggesting intracranial progression. Serum beta-hCG was 222 mIU/mL at that time. After discussion with the family, the same regimen was continued for one additional cycle, with the last treatment administered on October 8, 2024. Additional intracranial radiotherapy was initiated the following day at 3 Gy × 10 fractions.

During radiotherapy, the patient reported intermittent subxiphoid pain. Contrast-enhanced abdominal CT was recommended but declined. On October 22, after completion of radiotherapy, he developed abdominal pain and distension that improved with symptomatic management. On October 26, he experienced sudden severe abdominal pain and shock. CT revealed intra-abdominal hemorrhage and hepatic metastases. Despite emergency resuscitation, the patient died.

## Materials and methods

### Next-generation sequencing

Comprehensive genomic profiling was performed by Geneseeq Technology Inc. using a targeted panel covering 437 cancer-related genes. The assay included exonic regions, fusion-associated introns, alternative splicing regions, and selected microsatellite loci, with a total target region of approximately 1.53 Mb. The analysis assessed single-nucleotide variants, small insertions and deletions, gene fusions, copy-number variations, microsatellite instability status, and tumor mutational burden.

### Literature search and eligibility criteria

**Figure 3:**
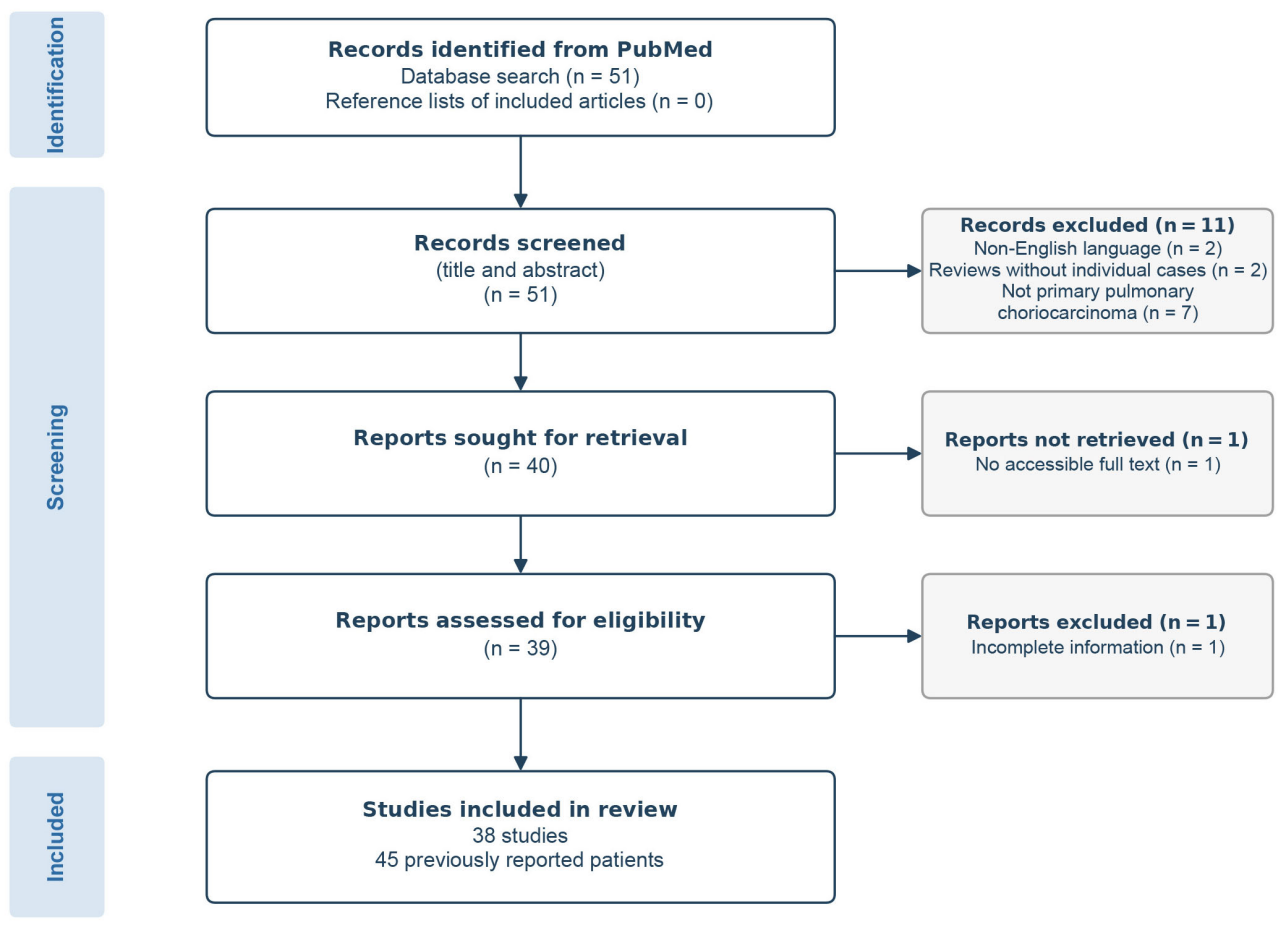
PRISMA 2020 flow diagram of study selection for the systematic review. Of 51 records identified through the PubMed search, 13 were excluded (2 non-English articles, 2 review articles without individual cases, 7 reports not describing primary pulmonary choriocarcinoma, 1 report without accessible full text, and 1 report with incomplete information), leaving 38 studies that together contributed 45 previously reported patients.

This systematic review was conducted and reported in accordance with the Preferred Reporting Items for Systematic Reviews and Meta-Analyses (PRISMA) 2020 statement (4). A systematic literature search was performed in PubMed using the following search terms: (((primary pulmonary choriocarcinoma[Title/Abstract]) OR (primary choriocarcinoma of the lung[Title/Abstract])) OR (primary choriocarcinoma of lung[Title/Abstract])) OR (pulmonary choriocarcinoma[Title/Abstract]).The search was restricted to English-language articles indexed in PubMed and covered publications from January 1, 2001, to February 2, 2026, with the final search executed on February 2, 2026. PubMed was used as the sole database because it comprehensively indexes the case-based literature on this rare tumor; to minimize omissions, the reference lists of all included articles were additionally screened by hand for further eligible reports. A total of 51 records were identified. After title, abstract, and full-text screening, 13 records were excluded (2 non-English articles, 2 review articles without individual cases, 7 reports not describing primary pulmonary choriocarcinoma, 1 report without accessible full text, and 1 report with incomplete information), leaving 38 eligible articles that together described 45 previously reported patients. The study-selection process is summarized in a PRISMA flow diagram (**Figure 3**).

Eligible studies were case reports or case series describing pathologically confirmed PPC with available individual patient-level clinical information. To be eligible, a report had to provide, at a minimum, pathological confirmation of primary pulmonary choriocarcinoma together with individual patient-level data on demographics, treatment, and clinical outcome; reports lacking any of these essential elements were considered to contain incomplete information and were excluded. Reports were excluded if they were non-English publications, duplicate reports, review articles without individual cases, studies lacking accessible full text or essential data, or reports of pulmonary metastases from choriocarcinoma originating in another organ. Study screening and selection were carried out independently by two investigators (Y.L. and L.W.), who also extracted data independently using a standardized, predefined data-extraction form capturing demographic characteristics, initial symptoms, diagnostic findings, treatment modality, and clinical outcome; disagreements at any stage were resolved by discussion until consensus was reached. The methodological quality of the included case reports and case series was appraised with the framework proposed by Murad et al., which addresses selection, ascertainment, causality, and reporting (5). Because the eligible evidence consisted exclusively of case reports and small case series with marked clinical heterogeneity and inconsistent reporting, no quantitative meta-analysis was undertaken, and the findings were synthesized narratively.

## Results

### Genomic findings in the present case

Targeted next-generation sequencing identified multiple alterations involving tumor suppressor and cancer-associated genes, including loss-of-function or deleterious alterations in *TP53*, *STK11*, *KEAP1*, and *SMARCA4* (**Table 1**). The complete profile comprised nonsense mutations in *STK11* (p.E223*) and *SMARCA4* (p.E365*); missense mutations in *TP53* (p.H179R), *KEAP1* (p.G417V), *APC* (p.P2522Q), *DOT1L* (p.I287V), *LRP1B* (p.H92Y), and *SDHA* (p.D38H); and a *CRIPT*-*CD74* gene fusion. The tumor was microsatellite stable, with no mismatch-repair gene alterations and a low tumor mutational burden (9.3 mutations/Mb). This predominantly tumor-suppressor profile, together with the patient’s long smoking history, is more consistent with a lung-carcinoma-like molecular background than with the chemotherapy-sensitive biology of gestational choriocarcinoma. However, because these data derive from a single patient, they should be regarded as hypothesis-generating and do not establish a general molecular signature of PPC or permit any genotype-treatment inference.

**Table 1:** Genomic alterations identified by next-generation sequencing.

Gene	Variant description	Mutation type	Plasma allele frequency (%)*	Tissue allele frequency (%)
*STK11*	Exon 5 nonsense mutation p.E223*	c.667G>T (p.E223*)	10.74	8.62
*TP53*	Exon 5 missense mutation p.H179R	c.536A>G (p.H179R)	14.76	10.09
*SMARCA4*	Exon 6 nonsense mutation p.E365*	c.1093G>T (p.E365*)	9.51	6.33
*APC*	Exon 16 missense mutation p.P2522Q	c.7565C>A (p.P2522Q)	8.52	3.20
*CD74*	*CRIPT*-*CD74* gene fusion	*CRIPT*:exon3~*CD74*:3’UTR	3.61	2.45
*DOT1L*	Exon 11 missense mutation p.I287V	c.859A>G (p.I287V)	9.74	3.85
*KEAP1*	Exon 3 missense mutation p.G417V	c.1250G>T (p.G417V)	10.24	6.25
*LRP1B*	Exon 3 missense mutation p.H92Y	c.274C>T (p.H92Y)	4.09	5.74
*SDHA*	Exon 2 missense mutation p.D38H	c.112G>C (p.D38H)	5.74	5.69

*Allele frequencies are reported as provided by the targeted sequencing assay.

### Systematic review findings

Most choriocarcinomas originate from the gonads, including the ovaries and testes. Extragonadal choriocarcinoma is rare and usually occurs along midline structures such as the mediastinum, retroperitoneum, pineal gland, and skull base (6). Although pulmonary metastases from choriocarcinoma are relatively common, PPC is exceedingly rare and has been associated with poor outcomes (7). The 38 eligible articles comprised 45 PPC patients (**Supplementary Table 1**). Representative reports published during the past three years are summarized in **Table 2**. Nineteen patients (42.2%) were male and 26 (57.8%) were female, with a median age of 44 years. Chemotherapy and surgery were each reported in 24 patients (53.3%), and surgery combined with chemotherapy was the most frequent treatment strategy (15/45, 33.3%). Immunotherapy was reported in 4 patients (8.9%), and targeted therapy in 1 patient (2.2%).

**Table 2:** Representative English-language PPC reports published during the past three years.

Patient no.	Author, year	Age (years)	Sex	Therapy	Outcome	Initial symptoms
01	Takahama, 2024 (11)	46	Male	Resection	Died 28 days after surgery	Cough, hemoptysis, dyspnea
02	Kaushik et al., 2024 (12)	34	Female	Targeted therapy and chemotherapy	Alive after 9 months without recurrence	Left-sided chest pain, dyspnea, weight loss, and anorexia
03	Okoli et al., 2024 (13)	32	Female	Resection and chemotherapy	beta-hCG normalization; PET-CT negative	NA
04	Abu Aljaaz et al., 2024 (14)	44	Female	Chemoradiotherapy followed by combined chemotherapy	Metastatic lesions achieved complete remission with marked beta-hCG reduction	Amenorrhea, headache, confusion
05	Devos et al., 2024 (15)	65	Male	Chemotherapy and immunotherapy	beta-hCG normalized; pulmonary lesions achieved PR	Bilateral gynecomastia, decreased libido, agitation, cough
06	Zhang et al., 2024 (16)	63	Female	Chemotherapy	NA	Recurrent postmenopausal vaginal bleeding
07	Liang et al., 2025 (8)	67	Male	Chemotherapy plus immunotherapy	Right lung mass decreased on CT after 4 months; no recurrence after 12 months	Cough, expectoration, hemoptysis
08	Yeung et al., 2025 (17)	69	Male	NA	Died 1 month after diagnosis	Dyspnea
09	Lu et al., 2025 (18)	73	Male	Cranial radiotherapy, chemotherapy, and radiotherapy for lung and liver lesions	beta-hCG level decreased	Progressive right limb numbness and weakness
10	Pan et al., 2026 (19)	60	Male	Chemotherapy plus immunotherapy	beta-hCG and CYFRA21–1 normalized; primary and metastatic lung lesions decreased	Cough, chest pain, dyspnea

NA, not available; PPC, primary pulmonary choriocarcinoma; PR, partial response.

Presenting manifestations were heterogeneous. In the pooled cases, cough and hemoptysis were the most frequent initial symptoms (24/45, 53.3%; **Supplementary Table 2**). In male patients, elevated hCG may cause feminizing manifestations such as gynecomastia, testicular atrophy, and decreased libido (8). PPC is also prone to early metastasis, recurrence, and choriocarcinoma syndrome, a hemorrhagic complication associated with high mortality (9). Diagnosis is difficult because early symptoms and imaging findings are nonspecific; on chest CT, PPC may appear as pulmonary nodules or masses and can be misclassified as other pulmonary diseases (10). Diagnostic confirmation generally requires exclusion of a primary gonadal lesion, pathological evidence of choriocarcinoma in the lung, and supportive serum or urinary beta-hCG dynamics (2). In the present case, markedly elevated serum hCG, absence of a gonadal primary tumor on ultrasonography, pathological and immunohistochemical findings, and post-treatment hCG decline supported the diagnosis of PPC.

## Discussion

This case illustrates the diagnostic and therapeutic challenges of metastatic PPC. The patient presented with brain metastases and markedly elevated serum beta-hCG, progressed after platinum-taxane chemotherapy, and then achieved prolonged extracranial disease control with an etoposide, capecitabine, and camrelizumab regimen combined with intracranial radiotherapy. The literature review supports the same clinical pattern: PPC is rare, often aggressive, and frequently managed by extrapolation from gestational choriocarcinoma, germ cell tumors, or lung cancer rather than from disease-specific trials. This case therefore adds to emerging case-based evidence on chemoimmunotherapy for metastatic PPC, while also documenting a distinct targeted-sequencing profile.

The available literature suggests that treatment outcomes depend strongly on resectability and disease extent. Cao et al. reported that sex, age, hemoptysis, metastasis status, and treatment modality were associated with survival, with surgery plus chemotherapy emerging as an independent prognostic factor (2). This evidence supports aggressive local therapy when disease is localized and operable, but it offers limited guidance for metastatic PPC. Recent case reports have described responses to immune checkpoint inhibitor-containing regimens, including nivolumab plus ipilimumab with chemotherapy (20), carboplatin, etoposide, and pembrolizumab (15), BEP chemotherapy plus tislelizumab (19), and sintilimab-based combination therapy (8). Molecularly profiled lung cancer with choriocarcinoma features has also been reported to respond to chemoimmunotherapy followed by local consolidation radiotherapy (21). The disease control observed in our patient is consistent with these reports but differs by the use of a camrelizumab-containing etoposide-capecitabine regimen after progression on platinum-taxane therapy. Because capecitabine, etoposide, camrelizumab, and repeated intracranial radiotherapy were administered in close sequence, this single case cannot establish the independent contribution of immune checkpoint inhibition or define a preferred regimen.

The genomic profile of this case adds to the limited molecular literature on PPC. Prior reports have identified alterations such as *TP53*, *NRAS*, *FGFR1*, and *EGFR* exon 20 mutations in individual PPC patients (3, 22), and another male PPC case was reported to harbor alterations involving *STK11* and *SMARCA4* (23). A molecularly profiled lung tumor with choriocarcinoma features further highlighted the diagnostic value of genomic and transcriptomic data in distinguishing lung-origin tumors from gestational or germ-cell choriocarcinoma (21). By contrast, whole-exome sequencing of gestational choriocarcinoma has shown frequent chromatin-remodeling alterations and uncommon *TP53* mutations (24). The presence of alterations involving *TP53*, *STK11*, *KEAP1*, and *SMARCA4* in our patient is therefore consistent with the hypothesis that PPC can be biologically distinct from gestational choriocarcinoma. However, *KEAP1* has not been established as a recurrent PPC alteration; its main interpretive context currently comes from non-small cell lung cancer, where *STK11* and *KEAP1* alterations have been associated with aggressive biology and less favorable outcomes with standard immunotherapy-based approaches (25).In keeping with this, the low tumor mutational burden and microsatellite-stable status of the present tumor do not represent a classically immunotherapy-favorable profile; together with the fact that PD-L1 expression was not assessed, this reinforces that the extracranial disease control observed cannot be attributed to a conventionally favorable immunotherapy biomarker profile. Given the patient’s long smoking history and tumor-suppressor alteration pattern, these findings raise the possibility of a lung-carcinoma-like molecular background with trophoblastic differentiation, but they remain hypothesis-generating and do not prove treatment sensitivity or resistance.

Several limitations should be emphasized. First, this report describes a single patient, so treatment response cannot be separated from disease heterogeneity, radiotherapy effects, and natural history. Second, the systematic review was restricted to English-language PubMed reports and was descriptive rather than meta-analytic. Third, published PPC cases vary in diagnostic workup, staging, treatment details, and follow-up duration, limiting direct comparison across reports. Finally, the genomic results are hypothesis-generating and require validation in larger multicenter datasets or rare-tumor registries.

## Conclusion

PPC is a rare and aggressive disease that appears clinically distinct from gestational choriocarcinoma and, on the basis of the present single case, may also be molecularly distinct. In this metastatic male PPC case, a camrelizumab-containing etoposide-capecitabine regimen with intracranial radiotherapy was associated with prolonged extracranial disease control after progression on first-line chemotherapy. Given that chemotherapy, immune checkpoint inhibition, and radiotherapy were administered concurrently, the independent contribution of immunotherapy to this outcome cannot be established. Because the evidence remains case-based and the genomic findings are hypothesis-generating, collaborative registries and multicenter molecular studies are needed to define the genomic landscape of PPC and to evaluate rational multimodal treatment strategies for this rare malignancy.

## Data Availability

The datasets presented in this study can be found in online repositories. The names of the repository/repositories and accession number(s) can be found in the article/**Supplementary Material**.
