# Supplementary material for: Clinicopathological and molecular characteristics of primary pulmonary choriocarcinoma: a case report and systematic review
Source: Front Oncol. 2026 Jun 30;16:1884668. doi: 10.3389/fonc.2026.1884668 (PMC13365297; doi:10.3389/fonc.2026.1884668)
Supplement: Supplementary file 3 [file Table3.docx]

**Supplementary Table S1. PRISMA 2020 Checklist**

| **Section / Topic** | **#** | **Checklist item** | **Location in manuscript** |
| --- | --- | --- | --- |
| **TITLE** |  |  |  |
| Title | **1** | Identify the report as a systematic review. | Title (“… A Case Report and Systematic Review”). |
| **ABSTRACT** |  |  |  |
| Abstract | **2** | See the PRISMA 2020 for Abstracts checklist. | Abstract. |
| **INTRODUCTION** |  |  |  |
| Rationale | **3** | Describe the rationale for the review in the context of existing knowledge. | Introduction, paragraphs 1–2. |
| Objectives | **4** | Provide an explicit statement of the objective(s) the review addresses. | Introduction, final paragraph. |
| **METHODS** |  |  |  |
| Eligibility criteria | **5** | Specify the inclusion and exclusion criteria and how studies were grouped. | Materials and Methods – Literature Search and Eligibility Criteria. |
| Information sources | **6** | Specify all databases/sources and the date each was last searched. | Methods – PubMed only; last search 2 February 2026; reference lists of included articles hand-searched. |
| Search strategy | **7** | Present the full search strategy for all databases. | Methods – full Boolean search string reported verbatim. |
| Selection process | **8** | Specify methods for screening and how many reviewers, working independently. | Methods – independent title/abstract and full-text screening by two reviewers; consensus. Figure 3. |
| Data collection process | **9** | Specify methods for data collection and number of reviewers. | Methods – independent extraction by two reviewers using a standardized, predefined form. |
| Data items | **10a** | List and define all outcomes for which data were sought. | Methods (clinical outcome); Table 2. |
| Data items | **10b** | List and define all other variables for which data were sought. | Methods (demographics, symptoms, diagnosis, treatment); Table 2. |
| Study risk of bias | **11** | Specify methods used to assess risk of bias, including tools used. | Methods – appraised with the Murad et al. tool for case reports/series (Ref 25). |
| Effect measures | **12** | Specify the effect measure(s) used in synthesis/presentation. | Not applicable – narrative synthesis; no quantitative effect measures (case-level data). |
| Synthesis methods | **13a–13f** | Describe processes for synthesis (eligibility, tabulation, methods, heterogeneity, sensitivity). | Methods – narrative synthesis; no meta-analysis owing to small numbers, heterogeneity and incomplete reporting. |
| Reporting bias assessment | **14** | Describe methods to assess risk of bias due to missing results. | Not applicable – no quantitative synthesis. |
| Certainty assessment | **15** | Describe methods to assess certainty in the body of evidence. | Not applicable – narrative synthesis of case-level data. |
| **RESULTS** |  |  |  |
| Study selection | **16a** | Report numbers screened, included and excluded, ideally with a flow diagram. | Figure 3 (PRISMA flow diagram); Methods. |
| Study selection | **16b** | Cite studies that met many but not all inclusion criteria, with reasons. | Figure 3 and Methods – 13 records excluded with reasons. |
| Study characteristics | **17** | Cite each included study and present its characteristics. | Table 2; Results – Systematic Review Findings. |
| Risk of bias in studies | **18** | Present assessments of risk of bias for each included study. | Discussion (limitations) – included reports are uncontrolled case reports/series (Murad appraisal). |
| Results of individual studies | **19** | Present summary statistics/results for each study. | Table 2 (individual patient-level data). |
| Results of syntheses | **20a–20d** | Summarize characteristics and results of the syntheses. | Results – Systematic Review Findings (narrative). |
| Reporting biases | **21** | Present assessment of risk of bias due to missing results. | Not applicable – no quantitative synthesis. |
| Certainty of evidence | **22** | Present assessments of certainty for each outcome. | Not applicable – narrative synthesis of case-level data. |
| **DISCUSSION** |  |  |  |
| Discussion | **23a** | Provide a general interpretation of the results in context. | Discussion, paragraphs 1–3. |
| Discussion | **23b** | Discuss limitations of the evidence included in the review. | Discussion – limitations paragraph. |
| Discussion | **23c** | Discuss limitations of the review processes used. | Discussion – English-language/PubMed restriction; descriptive synthesis. |
| Discussion | **23d** | Discuss implications for practice, policy and future research. | Discussion and Conclusion – need for registries and multicenter molecular studies. |
| **OTHER INFORMATION** |  |  |  |
| Registration and protocol | **24a–24c** | Provide registration information / protocol / amendments. | Not registered and no separate protocol; a priori eligibility criteria are reported in Methods. |
| Support | **25** | Describe sources of financial/non-financial support and role of funders. | Funding section. |
| Competing interests | **26** | Declare any competing interests. | Conflict of Interest section. |
| Availability of data, code | **27** | Report which materials are publicly available and where. | Data Availability Statement – all data are within the article and supplementary material. |

*、*
